# One Call Away: Bilingual Teleassessment for Preschool and Elementary Children: A Systematic Review

**DOI:** 10.1111/1460-6984.70136

**Published:** 2025-10-11

**Authors:** Aleksandra Kappenberg, Ulla Licandro

**Affiliations:** ^1^ Department of Special Needs Education and Rehabilitation Carl von Ossietzky Universität Oldenburg Oldenburg Germany

**Keywords:** bilingual assessment, teleassessment, remote assessment, telepractice, bilingualism, elementary children, primary children, systematic review

## Abstract

**Background:**

Despite the growing need for language and communication assessments in both languages for bilingual children, there remains a shortage of bilingual speech and language therapists (SLTs). Teleassessment has emerged as a promising solution to address this gap, but there is a pressing need for a comprehensive understanding of its organisation, implementation and feasibility across children of different ages, language combinations and proficiency levels.

**Aims:**

This systematic review aims to synthesise the current studies on bilingual language and communication teleassessment for preschool and elementary‐aged children. Specifically, it focuses on the language skills assessed in teleassessments, the tools and technology used and the organisational and implementation factors associated with bilingual teleassessment.

**Methods:**

The review was conducted following PRISMA guidelines. A systematic search was performed across five electronic databases as follows: APA PsycInfo, CINAHL, Education Source, Medline and Web of Science. Data from the selected studies were extracted and categorised, with study quality assessed using the Quality Assessment with Diverse Studies (QuADS).

**Main Contribution:**

A total of seven studies met the inclusion criteria. The review found that bilingual teleassessment typically focused on assessing productive and receptive vocabulary and grammar, using standardised tests adapted for remote administration. Most assessments were conducted in hybrid formats, combining both tele‐ and face‐to‐face elements. The results showed that language skills assessed via teleassessment were generally comparable to those assessed in face‐to‐face settings, indicating the feasibility of bilingual teleassessment.

**Conclusions and Implications:**

While bilingual teleassessment offers a promising approach to supporting bilingual children, its application should be approached with caution due to the limited number of studies and small sample sizes. Future research should prioritise the development of standardised guidelines for their implementation and the creation of targeted training and networking opportunities for bilingual SLTs. This will help enhance the quality and accessibility of bilingual teleassessment services.

**WHAT THIS PAPER ADDS?:**

*What is already known on this subject*

- Telepractice has been studied for several decades, particularly during the COVID‐19 pandemic. However, research on bilingual teleassessment for children with varying ages, language constellations and competencies remains limited. The variability in study objectives and target populations has made it challenging to identify optimal methods for organising bilingual teleassessment in both research and clinical practice.

*What this paper adds to existing knowledge*

- This study contributes to the existing literature by synthesising and critically evaluating research on bilingual teleassessment. It provides a comprehensive overview of the language and communication skills assessed, the tools and technologies used and the organisational, implementation and feasibility considerations in bilingual teleassessment.

*What are the potential or actual clinical implications of this work?*

- Although research on bilingual teleassessment is still evolving, this review highlights several effective organisational methods, best practices and challenges. The findings suggest that, similar to monolingual teleassessment, bilingual teleassessment is generally comparable to face‐to‐face assessment. To further support evidence‐based decision‐making in bilingual teleassessment, future studies—both single‐case and large‐scale—along with further training and networking for bilingual and monolingual SLTs, are essential.

## 1 Introduction

A comprehensive assessment of a child's communicative repertoire across all languages is fundamental to targeted speech and language therapy (SLT) for bilingual children (Freeman and Schroeder 2022; Kohnert et al. 2020). Although many children require assessment in multiple languages, only a small proportion of SLTs worldwide are bilingual service providers (American Speech‐Language‐Hearing Association 2021; Bloder et al. 2023; Stankova et al. 2021). Consequently, bilingual SLTs are often not readily available locally. To address these resource limitations, children's language skills can be assessed via teleassessment. Research on teleassessment, particularly for children, has expanded during the COVID‐19 pandemic (Fong et al. 2021; Schmitt et al. 2022); however, existing studies vary in their aims and methodologies. In the specific area of bilingual teleassessment, studies have assessed language and communication skills in children of different ages, with or without language disorders, and across various language combinations. The diverse methods employed in organising teleassessment present both opportunities and challenges for decision‐making and identifying best practices. To address this research gap, this paper aims to synthesise the current state of knowledge on bilingual teleassessment for children through a systematic review.

### 1.1 Bilingual Children and Language Assessment

Bilingual children regularly engage with more than one language in their daily lives, regardless of the age at which they acquired these languages or their proficiency levels (McLeod et al. 2017). The development of individual language and communication skills, as well as the time required for language acquisition, is influenced by a combination of personal and environmental factors (Armon‐Lotem and Meir 2018). Consequently, bilingual children display diverse language learning profiles that cannot be equated with those of monolingual speakers (Bedore and Peña 2008). However, significant expressive and/or receptive difficulties in both of a child's languages (Paradis et al. 2021) may indicate developmental language disorder (DLD; Bishop et al. 2017), which is diagnosed in approximately 7.6% of children, making it one of the most common developmental disorders (Norbury et al. 2016). Working with children from diverse linguistic backgrounds presents particular challenges for SLTs, especially in culturally and linguistically appropriate assessment and therapy. Although bilingual children are as likely to be affected by DLD as their monolingual peers (Paradis et al. 2021), they are often misdiagnosed—either over‐ or underdiagnosed—leading to missed or incorrect DLD identification (Grimm and Schulz 2014; Kohnert et al. 2020).

To distinguish between language difference and DLD, assessment procedures designed for monolingual children are insufficient (Bedore and Peña 2008; Boerma and Blom 2017). A bilingual assessment process should gather information on both languages through linguistic measures of speech production and comprehension at all linguistic levels, including speech and sound production, vocabulary, grammar and communicative skills, alongside evaluating the learning process using dynamic assessment (Hemsley et al. 2014; Kohnert et al. 2020; Stow and Pert 2015). Another crucial aspect is collecting comprehensive background information, including current language exposure and usage, cultural and social developmental contexts and the child's language development history (Li'el et al. 2019; Paradis et al. 2021). To accurately capture the complexity of DLD, it is recommended to consider not only the underlying disorder and its contextual factors but also the child's functioning and communication in everyday life, as well as their activities and participation in social interactions (Thomas‐Stonell et al. 2013; World Health Organisation 2007; Wright Karem et al. 2019). Importantly, data should be collected not only from various measures but also from multiple sources—such as home, school and community—and interpreted within the individual child's environment (Kohnert et al. 2020; Paradis et al. 2021).

Although several approaches to bilingual assessment have been established, current research indicates that their implementation in practice remains challenging. For instance, Stankova et al. (2021) found in their survey across Europe, Turkey and Lebanon that bilingual children are still predominantly assessed and treated in their second or environmental language. A similar survey from German‐speaking countries revealed that, despite SLTs being aware of bilingual assessment and therapy approaches, these are rarely applied in practice (Bloder et al. 2021). Overall, there remains a shortage of bilingual SLTs capable of evaluating all of a child's languages during the assessment process (American Speech‐Language‐Hearing Association 2021; Stankova et al. 2021). This shortage is compounded by the immense number and diversity of children's languages. In Europe alone, numerous minority and migration languages are spoken alongside 24 official languages (Sasse and Milt 2024). Consequently, even available bilingual SLTs cannot cater to all bilingual children with their various language constellations and competencies. To address these limitations and ensure access to bilingual expertise, telepractice offers a viable solution.

### 1.2 Telepractice in Speech and Language Therapy

The terms *telehealth*, *telerehabilitation* and *telemedicine* refer to healthcare services delivered remotely (Tenforde et al. 2020). In SLT, the term *telepractice* has been specifically adopted (Alfano et al. 2024; Speech Pathology Australia 2022). Telepractice includes remote screening, assessment, intervention, consultation and educational services, facilitated by technologies such as computers, smartphones and internet‐based platforms (Alfano et al. 2024; American Speech‐Language‐Hearing Association n.d.). It forms an integral part of the broader e‐health framework (Speech Pathology Australia 2022) and can serve as the primary mode of service delivery or complement in‐person sessions in hybrid models (American Speech‐Language‐Hearing Association n.d.). Telepractice offers the flexibility to provide SLT services anytime and anywhere, particularly benefiting individuals with limited access, such as those in rural areas or among underserved and vulnerable populations. Additionally, telepractice is both cost‐effective and time‐saving for SLTs, children and their families, as it eliminates the need for long‐distance travel (Alfano et al. 2024; Molini‐Avejonas et al. 2015). However, its effective implementation depends on several prerequisites, including digital competence of SLTs and clients, appropriate hardware, stable internet connectivity, access to suitable assessment and therapy materials as well as compliance with legal and data protection regulations (American Speech‐Language‐Hearing Association n.d.; Deutscher Bundesverband für akademische Sprachtherapie und Logopädie 2022; Royal College of Speech and Language Therapists 2022; Speech Pathology Australia 2022).

Although telepractice has been studied for nearly three decades (e.g., Duffy et al. 1997), the COVID‐19 pandemic and resulting contact restrictions significantly accelerated the development and use of teleassessment for children. While not all methods and tools originally designed for face‐to‐face assessment can be seamlessly adapted to remote formats (Krach et al. 2020), some standardised instruments have been evaluated for their usability in both settings. Teleassessment has demonstrated feasibility in evaluating various linguistic and communicative competencies in children, including expressive and receptive vocabulary (Lund and Werfel 2022; Schmitt et al. 2022) and narrative skills (Magimairaj et al. 2022; Nelson and Plante 2022). Additionally, a recent systematic review by Alfano et al. (2024) examined seven studies involving language‐based teleassessment for preschool and elementary‐aged children. The authors concluded that teleassessment is a reliable approach for assessing monolingual children aged 4–13 years with language and developmental disabilities, showing comparable adequacy to traditional face‐to‐face assessments. However, while these studies provide valuable insights into the organisation, implementation and feasibility of teleassessment, their findings primarily pertain to monolingual assessment conducted in school or societal languages.

### 1.3 Teleassessment for Bilingual Children

Teleassessment tailored to bilingual children necessitates careful consideration not only of digital materials and logistical infrastructure but also of the assessor's expertise in the child's first or home language. To date, relatively few studies have explored which tools are most effective for assessing first language and communication skills via teleassessment, or how such assessments can be optimally organised. For example, Guiberson et al. (2015) examined the accuracy of teleassessment measures for screening first language abilities in Spanish‐speaking preschool children with and without DLD in the United States, employing a synchronous hybrid approach. The children completed a non‐word repetition task via iPads and videoconferencing software with caregiver support. Additional tasks—including story retelling, expressive language skills using standardised tests and a parent questionnaire—were conducted face‐to‐face. The results revealed small to moderate, yet statistically significant, correlations between the teleassessment and in‐person measures. The authors concluded that combining non‐word repetition with other assessment tools enhanced classification accuracy and produced clinically relevant results. More recently, Hamdani et al. (2024) presented a case study involving the identification of DLD in a 6‐year‐8‐month‐old Urdu–Cantonese‐speaking girl, using teleassessment tools administered in Urdu. For comparison, seven typically developing Urdu–Cantonese‐speaking children were also tested. The assessment covered areas such as narrative skills, receptive and productive vocabulary and non‐word and sentence repetition. A synchronous videoconferencing approach was employed, with shared screen functionality; participants were provided with a headset and microphone, and used either a computer, laptop or tablet. The testing procedures adhered closely to the original face‐to‐face protocols, with each image supplemented by a number to support remote delivery. The child suspected of having DLD performed below her age‐matched peers on most measures. While this study provides valuable insights into the design and content of home language teleassessment, it does not discuss the implementation process or the feasibility of such methods in detail.

The organisation of bilingual teleassessment requires additional resources and considerations, because both of the child's languages must be documented and interpreted. First and foremost, a bilingual SLT proficient in the same languages as the child is required. In cases where such an SLT is unavailable, input from caregivers (Dam and Pham 2023), or the involvement or professional interpreters and translators may be necessary (Ancell and Hopf 2022). Second, appropriate bilingual assessment materials must be carefully selected and implemented. Given the limited availability of tools with parallel versions across multiple languages, clinicians are often required to adopt multi‐method approaches (Freeman and Schroeder 2022). To establish the best practices in this area, Peña and Sutherland (2022) provided an overview of teleassessment for both monolingual and bilingual children, drawing on insights from a discussion forum initiated during the COVID‐19 pandemic. The authors summarised the findings of nine studies, seven of which focused specifically on the processes and challenges with transitioning from face‐to‐face to teleassessment formats. Three studies have examined bilingual children with and without (risk of) DLD, two of which conducted bilingual teleassessment. While these studies provide valuable insights into the organisation of bilingual teleassessment, their focus has been limited to assessments involving English and Spanish. Furthermore, as only selected language and communication skills were assessed via standardised tests, the applicability of these findings to other language combinations and linguistic competences remains limited.

To enhance decision‐making and improve the quality of bilingual teleassessment in both research and clinical practice, there is an urgent need for further development of best practice approaches, alongside critical examination of their implementation and feasibility. Last but not least, the sustainability of teleassessment methods developed during the COVID‐19 pandemic—or prior to it—represents a significant gap in the current research landscape. As demonstrated by Kwok et al. (2022), both SLTs and caregivers have expressed interest in long‐term, flexible models of teleassessment and teleintervention. Notably, parents of children with diagnosed speech or language disorders have shown interest in adopting telepractice services as a means of enhancing language support for their child, even when they are not yet familiar with such approaches (Fitton et al. 2017). Bilingual children, in particular, stand to benefit substantially from flexible and sustained teleassessment and intervention models, given the additional barriers they often face in accessing appropriate SLT services (Scharff Rethfeldt et al. 2023).

### 1.4 Research Gap and Rationale

In conclusion, the exact organisation, implementation and feasibility of bilingual teleassessment for preschool and elementary‐aged children remain under development. To make informed decisions regarding its applicability, best practices and challenges, a well‐founded overview is required of how bilingual teleassessment can be organised to assess the language and communication skills of children of different ages, language combinations and competencies. Given the variety of available diagnostic tools and potential technological framework conditions, their exact implementation and feasibility in telepractice must be critically examined. To address this research gap, a systematic review that identifies, categorises and evaluates the current state of research on bilingual teleassessment is urgently needed. The overall aim is to provide a robust overview that will support informed decision‐making and thereby improve the quality of services and research in the area of SLT.

### 1.5 Study Aims

The aim of this systematic review was to identify and synthesise current studies on bilingual language and communication teleassessment for preschool and elementary‐aged children. Specifically, this review sought to address the following key questions:

1. Which language and communication skills were assessed in the respective languages?
2. What types of assessment tools were used?
3. What technology framework conditions (e.g., hardware and internet access) were reported?
4. How was the teleassessment organised?
5. What are the main results regarding the implementation and feasibility of bilingual teleassessment?

## 2 Methods

This systematic review was conducted in accordance with the Preferred Reporting Items for Systematic Reviews and Meta‐Analyses (PRISMA; Page et al. 2021), which provides guidelines for systematic reviews in the form of a 27‐item checklist and revised flow diagrams. At the outset of the research process, the review was registered in the International Prospective Register of Systematic Reviews (PROSPERO; ID CRD42023446852).

### 2.1 Eligibility Criteria

To be included in this review, studies had to meet the following inclusion and exclusion criteria. Empirical qualitative, quantitative or mixed‐methods studies were included if they investigated bilingual teleassessment in the context of language and/or communication for preschool or elementary‐aged children, with or without disabilities or disorders (e.g., DLD). To reflect the technological advancements over the past decade, the search was limited to studies published from 2010 onwards. Additionally, only studies published in English and appearing in peer‐reviewed academic journals were considered. Excluded were non‐empirical papers, studies focusing on teleassessment in monolingual children or adults, studies published prior to 2010, those written in a language other than English, and those published in non‐peer‐reviewed journals. Existing systematic reviews and meta‐analyses were also not considered.

### 2.2 Search Strategy

To identify studies on bilingual teleassessment, a comprehensive search string was developed using keywords related to teleassessment, digital assessment tools, bilingualism and childhood (see Table 1). The search was primarily conducted across five relevant databases as follows: APA PsycInfo, CINAHL, Education Source, Medline and Web of Science. The initial search was performed in August 2023, covering studies published between 1 January 2010 and 18 August 2023. An update was conducted in June 2024, using the same keywords and databases. A total of 81 articles were screened during the update, resulting in one additional article meeting the inclusion criteria. All results were uploaded to EPPI‐Reviewer (Thomas et al. 2010), a web‐based tool for managing systematic reviews.

**TABLE 1:** Search string.

Databases	Search string	Search in
APA PsycInfo CINAHL Education Source Medline Web of Science	(remote* OR digital* OR virtual* OR online* OR video* OR distance* OR web* OR internet* OR tele* OR ehealth) **NEAR/5** (assess* OR evaluat* OR measur* OR test* OR diagnos* OR elicit* OR screening* OR identif*) **AND** (bilingual* OR multilingual* OR “heritage language” OR “home language” OR “first language*” OR “culturally diverse” OR “linguistically diverse” OR “second language*” OR “dual language*” OR “language learn*”) **AND** (child* OR toddler* OR preschool* OR kindergarten* OR “elementary school” OR “primary school”)	Title, abstract, keywords

Additionally, the reference lists of the seven included papers, as well as the tables of contents of eight relevant scientific journals (see Appendix 1), were manually searched for further studies that met the inclusion criteria.

### 2.3 Quality Assessment

The quality of the seven studies included was assessed using the Quality Assessment with Diverse Studies (QuADS; Harrison et al. 2021). QuADS is specifically designed to evaluate the methodological and reporting quality of qualitative, quantitative, and mixed‐methods studies in systematic reviews related to health services. It comprises 13 criteria addressing various aspects of study design, rationale, result presentation and critical discussion. Each criterion is rated on a scale from 0 to 3 points. QuADS dispenses with fixed cut‐off values and instead encourages a contextualised discussion of study quality (Harrison et al. 2021). The studies were rated between 28 and 35 out of a maximum of 39 points and achieved the highest scores in six categories, including the rationale for the choice of data collection tool(s). The lowest scores were observed in the category ‘recruitment data provided’ (see Appendix 2).

### 2.4 Data Extraction and Narrative Synthesis

Data extraction involved collecting the following information from each study: (1) study details and objectives, (2) participant information, (3) examiner information, (4) language and communication skills assessed, (5) technology framework conditions, (6) types of assessment tools used, (7) organisation of the teleassessment and (8) implementation and feasibility outcomes. When a study included a teleintervention alongside teleassessment (e.g., McLeod et al. 2022), the intervention component was excluded from the data extraction process. The extracted data were then synthesised narratively (Popay et al. 2006), analysing commonalities and differences across studies.

### 2.5 Reliability

The first author conducted the primary abstract screening, with 10% of abstracts double‐screened by the second author for consensus. Both authors independently screened all full texts, carried out the quality assessments, and extracted core variables—including language and communication skills assessed, teleassessment organisation and feasibility. Any discrepancies at all stages were resolved through discussion, with the final results reflecting these consensus decisions.

### 2.6 Use of Artificial Intelligence Generated Content (AIGC)

The authors utilized ChatGPT to assist with language refinement while preparing this work. Following its use, the authors carefully reviewed and revised the material as necessary and accept full responsibility for the final content of the publication.

## 3 Results

**FIGURE 1:**
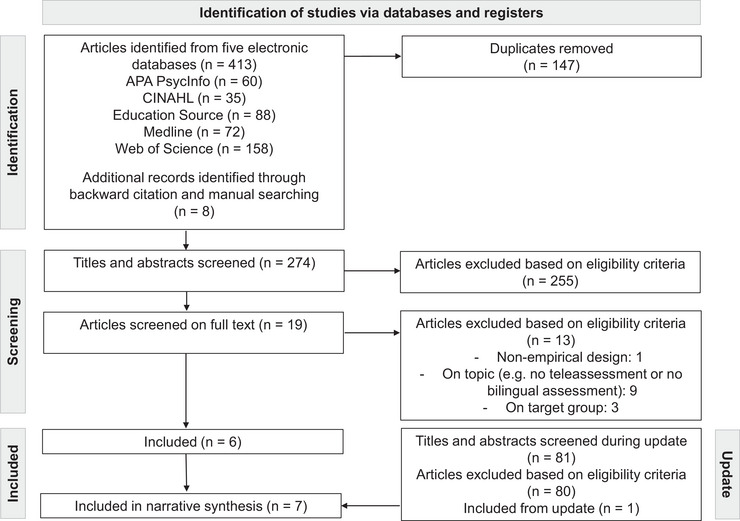
Preferred Reporting Items for Systematic Reviews and Meta‐Analyses (PRISMA; Page et al., 2021) flow diagram.

The initial search yielded a total of 413 results (see Figure 1). In the first step, 147 duplicates were removed, leaving 274 studies for title and abstract screening. Based on the eligibility criteria outlined earlier, 255 studies were excluded. The remaining 19 studies were then assessed in full text. Thirteen studies were excluded due to non‐empirical design, irrelevant topic (e.g., no teleassessment or no bilingual assessment), or incompatible target groups (e.g., children aged 0–2 years, adults). The final selection consisted of six studies, with one additional study identified during the update, resulting in a total of seven studies that met all inclusion criteria.

### 3.1 Study Characteristics and Aims

Six of the seven studies were conducted in English‐speaking countries, including the United States (e.g., Castilla‐Earls et al. 2022; see Table 2), in collaboration with the United States (e.g., Kokotek et al. 2024) and Australia (e.g., Yang et al. 2022). One study was conducted in Europe (Eikerling et al. 2023).

**TABLE 2:** Study overview.

	Authors, year and country	Study aims	Participants	Language and communication skills assessed	Type of assessment tools	Technology framework conditions	Organisation of teleassessment
1	Castilla‐Earls et al. (2022), USA	Effects of delivery method, impact of time, home language and diagnosis on children's receptive language scores	*N* = 89 (40 girls; *n* = 32 with DLD) Age: 47–98 months *M*_Age _= 5;9 years (SD = 12 months) Spanish‐ and English‐speaking (sequential or simultaneous bilinguals)	Receptive vocabulary	Standardised language tests adapted for teleassessment	Laptops/PCs with headphones and microphones	Teleassessment via videoconference Images from the test‐books were shown to the children with an extra HD camera. Children were asked to identify the number of the image/word selected.
2	Eikerling et al. (2023), Italy	Assessing the construct, convergent validity and diagnostic accuracy of tasks from the language screening platform	*N* = 36 (16 girls; *n* = 16 with DLD, *n* = 11 at risk of DLD) Age: 49–76 months *M*_Age _= 64.50 months (SD = 7.87) Spanish‐ and Italian‐speaking (early‐sequential or simultaneous bilinguals; Italian exposure for min. two years)	Dynamic novel word learning Non‐word repetition Receptive grammar (grammatical correctness at the syntactical level) Receptive vocabulary (verb comprehension)	Online platform for language screening Caregiver questionnaires	Laptops and PCs	Teleassessment via videoconference Screening administration through the link share Child's supporter managed the tasks using the screening platform. Children chose the answers via touch screen or by mouse click (if not possible, mouse clicking by supporter).
3	Pratt et al. (2022), USA	Feasibility of remote testing using child assessments originally validated for face‐to‐face testing Comparing the performance of children across face‐to‐face and remote condition	*N* = 10 (5 girls, all children TD) Age: 49–101 months *M*_Age _= 5;11 years (SD = 1;4) Spanish‐ and English‐speaking (5 simultaneous bilinguals, 5 monolingual English speakers)	Grammar Narrative skills (storytelling and retelling) Non‐verbal reasoning Semantics	Standardised language tests adapted for teleassessment SLT interview about the testing experience Caregiver questionnaires	Computers with webcams and headphones Technology check (e.g., sitting position, ambient noise, size of the child's device)	Teleassessment via videoconference and Qualtrics survey Language tests digitalised in Qualtrics and divided into two equal versions SLT and child in two separate rooms, both with a computer, webcam and headphones. Researcher observed and recorded the sessions via videoconference. Children chose receptive items by mouse click.
4	Dam and Pham (2023), USA	Feasibility and social validity of child teleassessment with the help of a caregiver	*N* = 21 (16 girls; all children TD) Age: 39–82 months *M*_Age _= 5.31 years (SD = 1.08) Vietnamese‐ and English‐speaking (sequential bilinguals, Vietnamese exposure since birth, *M*_AoO _= 2.59 years)	Narrative skills (storytelling and retelling) Language proficiency measures	Standardised language tests adapted for teleassessment Wordless picture books Caregiver questionnaires (inkl. social validity) Children's interview	Not mentioned	Teleassessment via videoconference All tasks were displayed through PowerPoint slides via ZOOM. Instructions were pre‐recorded and written in Vietnamese. After each session, SLT administered one additional language proficiency measure (in English or Vietnamese) as well as a child and a caregiver questionnaire.
5	Yang et al. (2022), Australia	Differences between bilingual preschoolers' narratives across languages and within each language Associations between bilingual preschoolers' narratives and their individual factors	*N* = 20 (15 girls; all children TD) Age: 46–76 months *M_Age_* = 4;11 years (SD = 8.7 months) Mandarin‐ and English‐speaking (sequential bilinguals; Mandarin exposure at home, *M*_AoO = _22 months, SD = 8.5 months)	Narrative skills (story retelling)	Wordless picture books (pre‐recorded) Caregiver questionnaire Caregiver language activities diary	Not mentioned	Teleassessment via videoconference A set of wordless pictures appeared on screen one by one with pre‐recorded audio. Children were asked to retell the story in their own words.
6	Kokotek et al. (2024), Jamaica and USA	Psychometric merits and stability of assessments used to measure communicative participation, functional speech intelligibility and speech production accuracy Characterisation of relationships among measures of communicative participation and speech intelligibility in the COVID‐19 milieu Differences in communicative participation, functional speech intelligibility and speech production accuracy of TD bilingual Jamaican preschoolers and those with fSSDs in COVID‐19 milieu compared to baseline data from before the pandemic	*N* = 30, 10 girls (*n* = 9 with fSSDs; *n* = 21 TD) Age: 43–74 months *M*_Age _= 53 months (SD = 1.14) Jamaican Creole‐ and English‐speaking (simultaneous bilinguals) Baseline from before the pandemic *N* = 265 (143 girls; *n* = 39 with fSDDs, *n* = 226 TD)	Speech production accuracy (percentage of consonants, vowels and phonemes correct, word‐level responses with articulation and phonology subtests) Communicative participation and functional speech intelligibility	Standardised language tests adapted for teleassessment Caregiver interview	Preferably PCs or laptops; tablets and smartphones also allowed	Teleassessment via videoconference Caregivers completed an interview in relation to children's communicative participation and functional speech intelligibility. Items in Jamaican‐Creole were played from an audio recording. Document camera was used to show picture stimuli in a standardised language test. A system of simple gestures was developed to communicate that a repetition was needed.
7	McLeod et al. (2022), Australia	Impact of intervention program on speech intelligibility, accuracy of speech sounds and vocabulary. Impact of intervention program on children's attitudes toward their home languages. Impact of intervention program on families' practicies regarding home language maintenance.	Intervention group *N =* 14 (7 girls; *n* = 7 at risk of SSD) Age: 46–79 months *M*_Age _= 59.93 (SD = 10.51) Control group *N *= 16 (9 girls; *n* = 11 at risk of SSD) Age: 24–94 months M_Age _= 61.88 months (SD = 25.52) Vietnamese‐ and English‐speaking (sequential or simultaneous bilinguals)	Speech production (percentage of Vietnamese and English consonants correct) Children's attitudes toward their languages	Standardised language tests adapted for teleassessment Child questionnaire Caregiver interview	Not mentioned	Teleassessment via videoconference

Abbreviations: AoO, age of onset; DLD, developmental language disorder; fSSDs, functionally defined speech sound disorders; M, mean; SD, standard deviation; SSD, speech sound disorders; TD, typically developing.

Five of the seven studies investigated research questions related to bilingual teleassessment, including its feasibility compared with face‐to‐face assessment (Castilla‐Earls et al. 2022; Pratt et al. 2022) and its implementation involving active caregiver involvement (Dam and Pham 2023). Additional research questions focused on the psychometric properties and stability of teleassessment instruments (Kokotek et al. 2024) and the validity and diagnostic accuracy of these tools for bilingual assessments (Eikerling et al. 2023). Two studies included teleassessment as part of their research but did not analyse it in detail (McLeod et al. 2022; Yang et al. 2022).

### 3.2 Participant Characteristics

Sample sizes across the included studies ranged from 10 to 89 participants, with more than half involving approximately 20 or fewer children. Five of seven studies focused on children aged approximately 4–6 years, who were either simultaneous or early sequential bilinguals. The majority of these children spoke English in combination with other languages, including Vietnamese, Spanish, Mandarin and Jamaican‐Creole. One study specifically examined children who grew up speaking Spanish and Italian.

Three of the seven studies included children diagnosed with, or at risk of DLD. Two studies focused on children with (suspected) speech sound disorders (SSDs) or functionally defined SDDs (fSDDs). The remaining studies exclusively examined typically developing children. Importantly, none of the studies included children with DLD associated with biomedical conditions, such as Down syndrome or hearing loss.

### 3.3 Examiners

In six of the seven studies, teleassessment was administered by bilingual SLTs, research assistants or linguists/psychologists in collaboration with the children's caregivers. In the study conducted by Dam and Pham (2023), caregivers were actively involved in recording the children's language skills, following detailed instructions. Notably, in the study by Pratt et al. (2022), eight of the SLTs were also the mothers of the children being assessed—a factor that warrants careful consideration in the interpretation of results.

### 3.4 Research Question 1: Language and Communication Skills

The studies examined a diverse range of linguistic and communicative skills across multiple languages. These included speech production accuracy (e.g., Kokotek et al. 2024), receptive vocabulary (e.g., Castilla‐Earls et al. 2022) and expressive vocabulary (e.g., Pratt et al. 2022), receptive grammar (e.g., Eikerling et al. 2023), and expressive grammar (e.g., Dam and Pham 2023). Narrative skills were also assessed, including story generation (e.g., Pratt et al. 2022) and retelling tasks (e.g., Yang et al. 2022). In addition, one study (Eikerling et al. 2023) assessed dynamic word learning and phonological working memory. Beyond linguistic measures, three studies explored broader communicative and experimental aspects, including children's participation (Kokotek et al. 2024), their attitudes toward languages (e.g., McLeod et al. 2022) and their experiences during assessment sessions (Dam and Pham 2023).

### 3.5 Research Question 2: Assessment Tools

Standardised language assessments adapted for teleassessment were the most frequently used tools in the studies involving direct child testing (e.g., Pratt et al. 2022). Two studies incorporated pre‐recorded wordless picture books to elicit narrative retelling and evaluated other language skills (Dam and Pham 2023; Yang et al. 2022). One study (Eikerling et al. 2023) employed an online multilingual language screening platform to document children's skills across different languages. In addition to direct assessments, several studies utilised questionnaires and interviews to gather supplementary data. More than half used caregiver questionnaires to collect information about the children's language backgrounds and communicative competencies (e.g., Yang et al. 2022). Two studies conducted caregiver interviews (e.g., McLeod et al. 2022), while one included an interview with an SLT regarding their experience with the teleassessment process (Pratt et al. 2022). One study administered a child questionnaire (McLeod et al. 2022), and another included a child interview (Dam and Pham 2023). Finally, Yang et al. (2022) recorded data on children's everyday language use using a caregiver‐maintained language activity diary.

### 3.6 Research Question 3: Technology Framework Conditions

More than half of the studies provided details on the hardware setup used for teleassessment. Three studies exclusively allowed participation via desktop or laptop computers (Castilla‐Earls et al. 2022; Eikerling et al. 2023; Pratt et al. 2022), with both Castilla‐Earls et al. (2022) and Pratt et al. (2022) explicitly required participants to use headphones. Notably, Castilla‐Earls et al. (2022) implemented a hardware loan program to support participants who lacked access to suitable devices and/or a stable internet connection. While Kokotek et al. (2024) preferred the use of desktop or laptop computers, their study also allowed participation via tablets and smartphones.

Kokotek et al. (2024) was the only study to systematically record and analyse the type of hardware used and internet speed. Approximately 60% of participants used desktop or laptop computers for assessments of English and Jamaican‐Creole skills. Smartphones were the second most commonly used device, accounting for over 20% of participation. Assessments were completed using built‐in microphones on the respective devices. The study also reported that approximately 40% of participants had internet speeds below the recommended minimum threshold. However, when telepractice conditions were included as a covariate in the analysis, no significant effects were found on the production of consonants, vowels or phonemes in either language.

Three of the seven studies did not specify hardware requirements or provide details or recommendations regarding internet speed (Dam and Pham 2023; McLeod et al. 2022; Yang et al. 2022).

### 3.7 Research Question 4: Organisation of Teleassessment

More than half of the reviewed studies employed a hybrid data collection approach, combining teleassessment with face‐to‐face assessment. Two studies were conducted entirely via telepractice (Dam and Pham 2023; Yang et al. 2022), while the remaining studies utilised a hybrid format. All studies used various videoconferencing platforms to assess children's language and communication skills, with most reporting that sessions or segments were audio‐ and/or video‐recorded. Bilingual teleassessments were typically conducted across one to four sessions, each lasting up to 60 min. With the exception of Castilla‐Earls et al. (2022), both of the children's languages were assessed within the same session.

Bilingual teleassessment was successfully implemented using a range of methods. Five studies digitised standardised language tests and modified stimuli for remote administration. In Castilla‐Earls et al. (2022) and Kokotek et al. (2024), a second camera was used to display the test book images to the children. Pratt et al. (2022) digitised all testing materials in Qualtrics, creating parallel versions for face‐to‐face and teleassessment formats. Children selected receptive items directly via mouse‐click; certain tasks were digitally analysed, while others were recorded and transcribed in Qualtrics. Eikerling et al. (2023) administered language screening through a shared link to the screening platform, where children selected responses via touchscreen or mouse click. Analysis of selected components was also conducted digitally.

To assess narrative skills, Dam and Pham (2023) presented tasks using PowerPoint slides via screen sharing. Following each session, additional language proficiency measures were collected, along with child and caregiver questionnaires. A similar protocol was followed by Yang et al. (2022), who used a set of wordless pictures with pre‐recorded audio. Kokotek et al. (2024) also conducted caregiver interviews, during which items in Jamaican Creole were played from audio recordings. Although McLeod et al. (2022) indicated the use of both teleassessment and hybrid models, they did not provide detailed descriptions of their implementation procedures.

### 3.8 Research Question 5: Implementation and Feasibility

The implementation of bilingual teleassessment demonstrated comparability to face‐to‐face assessment for several language skills. For example, Castilla‐Earls et al. (2022) reported that bilingual receptive vocabulary assessments administered via teleassessment were comparable to face‐to‐face assessments for bilingual children with and without DLD. Similarly, Pratt et al. (2022) found equivalent results for various language skills—including receptive and expressive grammar, and narrative skills—in typically developing mono‐ and bilingual children. Eikerling et al. (2023) evaluated the web‐based bilingual screening platform MuLiMi and found correlations between screening tasks assessing the same linguistic areas in two different languages, supporting the tool's construct validity. Moreover, significant correlations between remote screening tasks, standardised face‐to‐face assessments, and parental questionnaires provided additional evidence for the platform's convergent validity. Dam and Pham (2023) demonstrated that caregiver‐directed bilingual teleassessment yielded language samples comparable to those collected by SLTs. Kokotek et al. (2024) reported that communicative participation measures obtained via teleassessment remained stable for children with and without fSSDs. Although some variability in speech production accuracy was observed, the differences between groups were not statistically significant when telepractice was included as a covariate. Yang et al. (2022) and McLeod et al. (2022) focused on narrative skills and bilingual speech interventions delivered via telepractice, but did not provide detailed analyses of the implementation of teleassessment in their findings.

With regard to feasibility, the studies identified several best practice examples and challenges, particularly related to planning of assessment sessions, technology used and the participation of both caregivers and children (see Table 3). It is noteworthy that two studies (Castilla‐Earls et al. 2022; McLeod et al. 2022) were not specifically designed to compare teleassessment with face‐to‐face assessment, which limits the generalisability of their findings across different settings.

**TABLE 3:** Feasibility of bilingual teleassessment.

	Best practices	Challenges
Planning assessment sessions	– Development of structured and culturally adapted assessment protocols tailored for caregivers to ensure relevance and accessibility (Dam and Pham 2023) – Reduction of administrative and technological complexities to facilitate smoother implementation of teleassessments (Dam and Pham 2023) – Design of highly structured activities to provide clarity and maintain consistency throughout the assessment process (Dam and Pham 2023) – Provision of guidelines for families lacking access to recommended technological resources, ensuring equitable participation (Kokotek et al. 2024) – Implementation of engaging and motivating task designs (Eikerling et al. 2023), complemented by virtual rewards to sustain children's interest (Pratt et al. 2022) – Overestimating the number of sessions as a strategy to accommodate unforeseen changes (Pratt et al. 2022) – Assignment of a consistent examiner across all sessions to ensure reliability and familiarity in the assessment process (Pratt et al. 2022)	– Child distraction due to simultaneous testing and documentation, potentially affecting assessment performance (Pratt et al. 2022) – Feasibility limitations of certain measures (e.g., number of word productions) in online settings, requiring alternative assessment strategies (McLeod et al. 2022)
Technology	– Utilization of a second camera for displaying assessment materials (Castilla‐Earls et al. 2022; Kokotek et al. 2024) – Use of enlarged images and assignment of numbers to visual stimuli for clarity and ease of identification (Castilla‐Earls et al. 2022; Pratt et al. 2022) – Implementation of pre‐assessment technology checks to ensure optimal functioning of hard‐ and software (Pratt et al. 2022) – Allowing children to control the assessment interface using a mouse or touch screen to enhance engagement and activity (Pratt et al. 2022) – Encouragement of headset use to improve audio quality and minimise environmental distractions (Pratt et al. 2022) – Recommendations for improving internet connectivity, such as using a wired connection and disabling video to optimise bandwidth (Pratt et al. 2022) – Integration of pre‐recorded instructions and narrative retell tasks to streamline the assessment process and ensure consistency (Dam and Pham 2023) – Feasibility of conducting (speech sound) assessments without high‐fidelity equipment, with a cautionary approach to result in interpretation to account for potential limitations (Kokotek et al. 2024)	– Insufficient access to technical equipment among the families involved in the assessment (Castilla‐Earls et al. 2022; Kokotek et al. 2024) – Poor audio quality impacting the assessment process (Pratt et al. 2022; Yang et al. 2022), as well as environmental noise interference (Yang et al. 2022) – Unstable internet connections (Kokotek et al. 2024; Yang et al. 2022), leading to a misalignment between visual and auditory cues (Pratt et al. 2022) – Lack of dynamic elements in pre‐recorded audio demonstrations, affecting interactive assessment quality (Pratt et al. 2022)
Caregiver participation	– Benefits of facilitating children's involvement (Dam and Pham 2023; Eikerling et al. 2023; Pratt et al. 2022) – Viable option: caregiver as a direct assessment facilitator (Dam and Pham 2023)	– Challenges for SLTs in managing interruptions during teleassessment sessions (Yang et al. 2022)
Child participation	– Adjusting session duration based on age and developmental skills, with sessions lasting approximately 30 min for children aged 4–5 years, 45–55 min for children aged 7–9 years (Pratt et al. 2022)	– Challenges with code‐switching (Yang et al. 2022) – Difficulties performing tasks due to limited language exposure in both languages assessed (Dam and Pham 2023; Eikerling et al. 2023) – Difficulty remaining seated during sessions (Yang et al. 2022) – Decreased attention span (McLeod et al. 2022; Pratt et al. 2022) – Teleassessment challenges for younger children due to developmental demands of tasks involving number and colour selection, and mouse use (Eikerling et al. 2023; Pratt et al. 2022)

## 4 Discussion

This systematic review aimed to identify and summarise the current state of research on bilingual teleassessment for preschool and elementary‐aged children. Most studies focused on assessing receptive and expressive vocabulary and grammar, predominantly using standardised language tests adapted for remote administration. Where technological requirements were reported, participation was generally limited to desktop or laptop computers. Teleassessment was typically conducted in a hybrid format, and all studies employed videoconferencing platforms to support synchronous assessment.

Findings related to the implementation of bilingual teleassessment revealed high levels of comparability with face‐to‐face assessment across multiple language domains. Statistically significant correlations across assessment formats suggest that teleassessment methods can provide valid and reliable measures of bilingual children's language abilities. In addition, the review identified various best practices and feasibility considerations, including device access, session structure and the involvement of both children and their caregivers.

### 4.1 Language and Communication Skills

Although the majority of studies on bilingual teleassessment have focussed on assessing vocabulary and grammar (e.g., Castilla‐Earls et al. 2022; Pratt et al. 2022), several also explored other competences, such as narrative skills (e.g., Dam and Pham 2023; Yang et al. 2022), communicative participation (Kokotek et al. 2024) and learning potential within the framework of dynamic assessment (Eikerling et al. 2023). Many studies assessed multiple linguistic domains, providing a more comprehensive picture of children's skills across their two languages (e.g., Eikerling et al. 2023; Pratt et al. 2022). However, as noted in the scoping review by Wright Karem et al. (2019), there remains a tendency in the literature to prioritise linguistic competence over everyday communicative functioning. This limitation is also evident in the present review. While some studies have gathered information on the children's pragmatic development using parental questionnaires (e.g., Eikerling et al. 2023), to date, Kokotek et al. (2024) is the only study to have examined the detailed communicative participation of bilingual children via teleassessment. To build a more comprehensive understanding of bilingual language development—and to improve diagnostic accuracy—it is essential to assess not only formal language skills but also children's ability to engage in their everyday language environments, participate in age‐appropriate activities and interact socially (McLeod et al. 2022; Thomas‐Stonell et al. 2013; WHO 2007).

### 4.2 Assessment Tools

To assess children's bilingual abilities via telepractice, studies most frequently employed standardised language tests adapted for remote administration (e.g., Dam and Pham 2023; Pratt et al. 2022). Methods incorporating two (or more) parallel language versions were commonly used for this purpose. While the measurement accuracy of several standardised tests for monolingual teleassessment has been successfully validated (Lund and Werfel 2022; Magimairaj et al. 2022; Nelson and Plante 2022), equivalent versions tailored to bilingual assessment remain scarce (Freeman and Schroeder 2022). Since most standardised tests are designed and normed for monolingual children only, their application for bilingual children is highly debated, primarily due to the challenges associated with applying monolingual norm data to bilingual populations. This issue arises from the lack of comparability when using monolingual norms, which may not accurately reflect the linguistic diversity and specific needs of bilingual children (Thordardottir 2015). Hence, it is important to recognize that such methods may not be universally applicable to all bilingual populations that could potentially benefit from teleassessment. Overall, studies that employed non‐standardised procedures adapted for teleassessment should be interpreted with caution. Many studies also incorporated questionnaires and interviews to collect supplementary data on children's development and personal circumstances (e.g., Eikerling et al. 2023). However, study descriptions often lacked clarity as to whether the questionnaires were purpose‐built digital tools for teleassessment or traditional paper‐and‐pencil instruments.

### 4.3 Technology Framework Conditions

Although teleassessment requires careful consideration of technological conditions, only four of the seven studies provided detailed information on this aspect. To ensure adequate screen size, three studies (e.g., Eikerling et al. 2023) restricted participation to PCs or laptops. While this enhances procedural comparability, it may also contribute to the underrepresentation of children from low socio‐economic backgrounds. To address this issue, Kokotek et al. (2024) permitted the use of any digital device, accepting greater variability in data quality in favour of increased inclusivity. Notably, Kokotek et al. (2024) was the only study to record and analyse internet speed in the context of bilingual teleassessment. Similar to findings in monolingual teleassessment (Alfano et al. 2024), there is currently limited evidence regarding the impact of specific technological factors on the accuracy and quality of bilingual teleassessment administration.

### 4.4 Organisation of Teleassessment

Most of the reviewed studies implemented hybrid teleassessment approaches to evaluate children's competencies in both languages. All studies employed video conferencing platforms for synchronous communication with participants. Although Zoom is widely used for telepractice globally (McGill and Fiddler 2021), legal and data protection regulations vary significantly across regions (American Speech‐Language‐Hearing Association n.d.; Royal College of Speech and Language Therapists 2022; Speech Pathology Australia 2022). For instance, in Germany, the use of Zoom or Microsoft Teams for SLT telepractice is currently not permitted (Deutscher Bundesverband für akademische Sprachtherapie und Logopädie 2022).

Various methods were employed to adapt existing bilingual assessments for remote delivery. The use of a second camera by Castilla‐Earls et al. (2022) and Kokotek et al. (2024) enabled the display of test materials designed for face‐to‐face diagnostics. As standardised tests are unavailable in many languages (Boerma and Blom 2017; Eikerling et al. 2025), alternative methods such as language sampling (Dam and Pham 2023; Yang et al. 2022), and dynamic assessment (Eikerling et al. 2023; Freeman and Schroeder 2022) warrant further investigation for their applicability in teleassessment contexts. Additionally, pre‐recorded segments of the assessment (Kokotek et al. 2024; Yang et al. 2022) may be a viable option when the SLT does not speak the child's first language, allowing for subsequent analysis by bilingual interpreters. However, copyright issues must be taken into account when modifying and implementing assessment tools for telepractice.

### 4.5 Implementation and Feasibility

The implementation of bilingual teleassessment has demonstrated comparability to face‐to‐face assessments across a range of linguisitc skills and language constellations. While many procedures have demonstrated equivalence across settings (Alfano et al. 2024; see also the assessment tools section), the evidence base for bilingual teleassessment remains limited. Although the studies by Castilla‐Earls et al. (2022) and Pratt et al. (2022) contribute important findings on effective procedures for bilingual children, caution is advised in interpreting data obtained via teleassessment. Assessing bilingual language development requires specialised knowledge of bilingual acquisition and diagnostic processes (Boerma and Blom 2017; Paradis et al. 2021). Nevertheless, caregiver‐directed measures—when supported with clear guidance—can yield results comparable to those obtained by SLTs (Dam and Pham 2023). However, caregivers from lower socio‐economic backgrounds may struggle to provide such support, even when adequately instructed. The expansion of cooperation with professional interpreters and translators is therefore essential (Ancell and Hopf 2022), yet remains difficult to access for many SLTs (Eikerling et al. 2025).

The studies reviewed highlighted both best practices and persistent challenges, particularly with regard to the organisation of assessment sessions, technological requirements and engagement of both children and caregivers. Careful planning that ensures sessions are engaging while minimising administrative and technological complexity is crucial (Dam and Pham 2023; Eikerling et al. 2023; Pratt et al. 2022). While some studies have provided valuable insights into the cultural adaptation of assessment instruments (Dam and Pham 2023), this remains an area with notable research gaps—particularly in relation to specific contexts such as language combinations and personal migration experiences. Children generally require support—or at minimum, supervision—from caregivers to participate effectively in teleassessment. However, the role of adults and the type of support they provide remains an under‐researched area. While some caregivers prefer traditional face‐to‐face assessments (Campbell et al. 2024), others value telepractice services as a means of improving access to SLT services (Fitton et al. 2017). Despite concerns about children's participation—such restlessness (Yang et al. 2022) or reduced attention span during the sessions (McLeod et al. 2022; Pratt et al. 2022)—Campbell et al. (2024) found no differences in the frequency of child disruptions between tele‐ and face‐to‐face settings. Overall, the identification of best practices and challenges underscores the importance of intentional planning, technological support, and adaptation of the assessment environment to optimise the effectiveness of bilingual teleassessment.

### 4.6 Limitations

When interpreting the results, several limitations should be considered. Most studies were conducted in English‐speaking countries, such as the United States (e.g., Castilla‐Earls et al. 2022) and Australia (e.g., Yang et al. 2022), reflecting limited geographic and linguistic diversity. This concern, previously raised by Molini‐Avejonas et al. (2015) in the context of telepractice, is also frequently observed in bilingualism research (Wright Karem et al. 2019).

Moreover, the reviewed studies included heterogeneous groups of children with varying ages, language backgrounds and competencies. Only Castilla‐Earls et al. (2022) and Pratt et al. (2022) included children over the age of eight. The total number of studies was limited (*n*  =  7), and they differed substantially in sample size, objectives and methodology. To maintain the rigour of this systematic review, only peer‐reviewed studies were included, thereby excluding potentially relevant publications such as conference papers or dissertations. Additionally, this review focuses on a rapidly evolving research area, with numerous publications anticipated following the COVID‐19 pandemic. However, due to the complexity of the review process, no literature published after July 2024 was included. Despite multiple refinements and expert consultations to the search strategy, the specificity of search terms and language limitations may have excluded relevant studies.

The quality appraisal of the studies ranged from 28 to 35 points, indicating overall high quality. However, many studies scored low on criteria‐related recruitment transparency and stakeholder involvement. Notably, the lack of substantial consultation with stakeholders is concerning, as such engagement enhances the critical scrutiny and practical relevance of the research. Finally, the review was conducted by only two authors, increasing the risk of subjectivity in data selection, analysis and interpretation.

### 4.7 Implications

This review identified a range of effective procedures available for use by bilingual SLTs to assess children's language abilities via teleassessment. Future efforts should prioritise training and networking opportunities for bilingual SLTs, facilitating a clearer overview of language‐specific expertise and promoting collaboration with monolingual SLTs. Although bilingual SLTs served as examiners in most reviewed studies, their global availability remains limited (American Speech‐Language‐Hearing Association 2021; Bloder et al. 2023; Stankova et al. 2021), meaning that, even with telepractice, many bilingual children with language and communication disorders may not receive appropriate support. Involving caregivers and professional interpreters in the diagnostic process is essential (Ancell and Hoff 2022; Dam and Pham 2023; Eikerling et al. 2025). Despite the advantages of teleassessment, its implementation is not yet widely supported or funded in all countries. For instance, while certain therapy sessions can be conducted remotely in Germany, teleassessment is currently not approved (Deutscher Bundesverband für akademische Sprachtherapie und Logopädie 2022).

To effectively establish bilingual teleassessment for target groups with diverse language backgrounds and competencies, future research must include a wider range of languages and language combinations, including those spoken by minority groups. Studies should also include elementary‐aged children and those with diagnosed language disorders, such as DLD associated with biomedical conditions. In addition, researchers must systematically document technological conditions and integrate them into analyses. Given that up to two‐thirds of the world's school‐aged children globally lack reliable internet access at home (UNICEF 2020) and many families lack essential hardware, further studies should explore low‐resource assessment methods and implement technology loan programmes for participants (see Castilla‐Earls et al. 2022). Research into asynchronous and hybrid formats with minimal hardware and internet requirements is also needed. Beyond large‐scale randomised controlled studies, current systematic reviews and well‐documented case studies from SLT practice would provide valuable insights into the feasibility and effectiveness of bilingual teleassessment. These are essential for informing policy changes and establishing a robust evidence base for sustainable bilingual teleassessment.

## 5 Conclusion

This systematic review demonstrates that bilingual teleassessment—much like monolingual teleassessment—can yield results comparable to traditional in‐person assessment, highlighting its potential for expanding access to services for linguistically diverse populations. However, as the evidence base is still evolving, its implementation should be approached with caution, ensuring continuous evaluation of its feasibility, reliability and clinical effectiveness.

## Disclosure

The authors hereby declare that they are pursuing exclusively scientific and not commercial interests.

## Ethics Statement

The authors have nothing to report.

## Consent

The authors have nothing to report.

## Conflicts of Interest

The authors declare no conflicts of interest.

## Supporting information




**Supporting**: Appendix 1
**Supporting**: Appendix 2

## Data Availability

Data sharing is not applicable to this article as no datasets were generated during the current study.
