# Supplementary material for: One Call Away: Bilingual Teleassessment for Preschool and Elementary Children: A Systematic Review
Source: Int J Lang Commun Disord. 2025 Oct 11;60(6):e70136. doi: 10.1111/1460-6984.70136 (PMC12515270; doi:10.1111/1460-6984.70136)
Supplement: Supplementary file 1 — Supporting: Appendix 1 Supporting: Appendix 2 [file JLCD-60-0-s001.docx]

Appendix 1

List of scientific journals for manual screening of tables of contents:

1. International Journal of Language & Communication Disorders
2. International Journal of Speech-Language Pathology
3. Language, Speech, and Hearing Services in Schools
4. Journal of Speech, Language, and Hearing Research
5. Journal of Communication Disorders
6. Journal of Telemedicine and Telecare
7. Telemedicine and e-Health
8. American Journal of Speech and Language Pathology

Appendix 2

Ratings for the quality assessment (QuADS; Harrison et al., 2024)

| **QuADS Criteria (Harrison et al., 2021)** | Castilla-Earls et al. (2022) | Eikerling et al. (2023) | Pratt et al., (2022) | Dam & Pham (2023) | Yang et al. (2022) | Kokotek et al. (2024) | McLeod et al. (2022) |
| --- | --- | --- | --- | --- | --- | --- | --- |
| 1 Theoretical or conceptual underpinning to the research | 3 | 1 | 3 | 3 | 2 | 3 | 2 |
| 2 Statement of research aim/s | 3 | 3 | 3 | 3 | 3 | 3 | 3 |
| 3 Clear description of research setting and target population | 3 | 3 | 3 | 3 | 3 | 3 | 3 |
| 4 The study design is appropriate to address the stated research aim/s | 2 | 3 | 3 | 3 | 3 | 3 | 2 |
| 5 Appropriate sampling to address the research aim/s | 2 | 2 | 2 | 2 | 1 | 2 | 2 |
| 6 Rationale for choice of data collection tool/s | 3 | 3 | 3 | 3 | 3 | 3 | 3 |
| 7 The format and content of data collection tool is appropriate to address the stated research aim/s | 3 | 3 | 3 | 3 | 2 | 3 | 3 |
| 8 Description of data collection procedure | 3 | 3 | 3 | 3 | 3 | 3 | 3 |
| 9 Recruitment data provided | 0 | 1 | 1 | 1 | 0 | 2 | 2 |
| 10 Justification for analytic method selected | 3 | 3 | 3 | 3 | 3 | 3 | 3 |
| 11 The method of analysis was appropriate to answer the research aim/s | 3 | 3 | 3 | 3 | 3 | 3 | 3 |
| 12 Evidence that the research stakeholders have been considered in research design or conduct. | 0 | 0 | 3 | 3 | 0 | 1 | 1 |
| 13 Strengths and limitations critically discussed | 2 | 3 | 2 | 2 | 2 | 2 | 2 |
| **Total score (max. 39)** | **30** | **31** | **35** | **35** | **28** | **34** | **32** |
